# Remdesivir and Obeldesivir Retain Potent Antiviral Activity Against SARS-CoV-2 Omicron Variants

**DOI:** 10.3390/v17020168

**Published:** 2025-01-25

**Authors:** Lauren Rodriguez, J. Lizbeth Reyes Zamora, Dong Han, Jasmine Moshiri, Nadine Peinovich, Clarissa Martinez, Pui Yan Ho, Jiani Li, Thomas Aeschbacher, Ross Martin, Andrew Pekosz, John P. Bilello, Jason K. Perry, Charlotte Hedskog

**Affiliations:** 1 Gilead Sciences, Inc., Foster City, CA 94404, USA; 2 Department of Molecular Microbiology and Immunology, Johns Hopkins Bloomberg School of Public Health, Baltimore, MD 21205, USA

**Keywords:** SARS-CoV-2, COVID-19, Omicron variants, remdesivir, genotyping, phenotyping, Nsp12

## Abstract

As new SARS-CoV-2 variants continue to emerge, it is important to evaluate the potency of antiviral drugs to support their continued use. Remdesivir (RDV; VEKLURY^®^) an approved antiviral treatment for COVID-19, and obeldesivir (ODV) are inhibitors of the SARS-CoV-2 RNA-dependent RNA polymerase Nsp12. Here we show these two compounds retain antiviral activity against the Omicron variants BA.2.86, BF.7, BQ.1, CH.1.1, EG.1.2, EG.5.1, EG.5.1.4, FL.22, HK.3, HV.1, JN.1, JN.1.7, JN.1.18, KP.2, KP.3, LB.1, XBB.1.5, XBB.1.5.72, XBB.1.16, XBB.2.3.2, XBC.1.6, and XBF when compared with reference strains. Genomic analysis identified 29 Nsp12 polymorphisms in these and previous Omicron variants. Phenotypic analysis of these polymorphisms confirmed no impact on the antiviral activity of RDV or ODV and suggests Omicron variants containing these Nsp12 polymorphisms remain susceptible to both compounds. These data support the continued use of RDV in the context of circulating SARS-CoV-2 variants and the development of ODV as an antiviral therapeutic.

## 1. Introduction

New variants of SARS-CoV-2 have continuously emerged since the beginning of the global COVID-19 outbreak. These variants have the potential for increased transmission, enhanced antibody evasion, and reduced effectiveness of vaccines and therapeutics [1,2]. The Omicron variant (Pango lineage B.1.1.529) was first reported in South Africa in November 2021 and, by the end of December 2021, had quickly overtaken Delta as the most prevalent variant in the United States [3,4]. Since then, Omicron variants have been the predominant circulating variants of interest (VOIs) and variants under monitoring (VUMs) worldwide [5,6].

There remains a need for effective therapies to treat the emerging variants of SARS-CoV-2. Remdesivir (RDV; GS-5734; VEKLURY^®^) is an intravenously administered nucleotide analog prodrug approved to treat COVID-19 in hospitalized and nonhospitalized adult and pediatric patients [7]. Obeldesivir (ODV; GS-5245) is an oral prodrug of the parent nucleoside of RDV with the potential to be a simple twice-daily treatment regimen with limited drug–drug interactions [8,9]. Using different metabolic pathways, RDV and ODV generate the same active nucleoside triphosphate (NTP) that acts as an inhibitor of the SARS-CoV-2 RNA-dependent RNA polymerase (Nsp12) by covalently incorporating into the primer strand, thereby terminating chain elongation or resulting in template-dependent inhibition [9,10,11]. A defining characteristic of RDV is its efficacy against a broad range of viruses [12,13,14,15,16,17,18,19,20]; ODV has shown a similar antiviral profile [9,12,21,22].

As new SARS-CoV-2 variants are detected, genomic surveillance plays an important role in monitoring emergent mutations that could confer resistance to existing therapeutics. The Omicron variant is characterized by over 30 amino acid substitutions in the spike protein, which impact the efficacy of therapeutic monoclonal antibodies [23]. As an example, the Omicron variants BF.7, BQ.1, XBF, XBB.1.5, and CH.1.1 exhibited greater antibody escape compared with previously detected variants, and a variety of monoclonal antibodies failed to neutralize the Omicron variants XBB.1.16 and XBB.1.9.1 [24,25,26,27]. Vaccines were consequently updated to improve protection against these and other circulating sub-lineages [28,29,30]. More recently, the emergence of the Omicron variant BA.2.86, which has more than 30 mutations in its spike protein relative to its ancestral variant BA.2, raised global concern [31]. BA.2.86 rapidly evolved into the currently circulating JN.1 sublineage [32], which includes subvariants KP.2, KP.3, and LB.1 [33]. These variants have shown effective immune evasion [32,33,34], prompting recommendations to update vaccine compositions for 2024 to 2025 to target JN.1 [35]. While RDV and ODV target the conserved RNA-dependent RNA polymerase region, which has minimal genetic evolution compared with the spike protein [36,37], it is important to confirm their remaining potency against SARS-CoV-2 variants to support continued use in COVID-19 treatment and development. Here, we assess the antiviral activity of RDV and ODV against recent Omicron variants using clinical isolates or by introducing the variant-defining substitutions present in the replication complex into a replicon system. In addition, the antiviral activity of RDV and ODV against Nsp12 polymorphisms observed in public databases was assessed in the replicon system.

## 2. Materials and Methods

To evaluate the in vitro antiviral activity of RDV and ODV against the Omicron variants BA.2.86, BF.7, BQ.1, CH.1.1, EG.1.2, EG.5.1, EG.5.1.4, FL.22, HK.3, HV.1, JN.1, XBB.1.5, XBB.1.5.72, XBB.1.16, XBB.2.3.2, XBC.1.6, and XBF, a previously described SARS-CoV-2 anti-nucleoprotein enzyme-linked immunosorbent assay (ELISA) was utilized in A549-hACE2-TMPRSS2 cells (a549-hace2tpsa; InvivoGen, San Diego, CA, USA) [9,38]. A total of 3 × 10^4^ A549-hACE2-TMPRSS2 cells in 100 µL Dulbecco’s Modified Eagle Medium (DMEM; supplemented with 10% fetal bovine serum [FBS] and 1× penicillin-streptomycin) were seeded into each well of a 96-well plate and incubated overnight. The following day, the medium was aspirated, and 100 µL of DMEM containing 2% FBS was added to each well. In triplicate, 3-fold serial dilutions of RDV (5–0.002 µM) or ODV (50–0.02 µM) were added to each well for a final volume of 200 µL/well. Immediately after compound addition, cells were infected with 1.5 × 10^3^ plaque-forming units of the relevant SARS-CoV-2 variant diluted in 100 µL of DMEM supplemented with 2% FBS, resulting in a multiplicity of infection of 0.05. Plates were centrifuged for 1 min at 500× *g* and then incubated at 37 °C with 5% CO_2_ for 3 days, after which the medium was aspirated and cells were fixed with 100% methanol for 10 min at room temperature (RT). The methanol was removed, and plates were air dried for 10 min at RT, followed by 1 h of incubation with 100µL/well of blocking buffer (phosphate-buffered saline [PBS] with 10% FBS, 5% nonfat dry milk, and 0.1% Tween 20) for 1 h at 37 °C. The blocking buffer was then aspirated, and 50 µL of a 1:10,000 dilution of rabbit anti-SARS-CoV-2 nucleocapsid antibody (MA5-36086 [4Z6T2]; Invitrogen, Waltham, MA, USA) in a blocking buffer was added and incubated for 2 h at 37 °C. Plates were washed 4 times with 200 µL/well of PBS containing 0.1% Tween 20 prior to the addition of 50 µL/well of horseradish peroxidase conjugated goat anti–rabbit IgG (GtxRb-003-FHRPX; ImmunoReagents, Raleigh, NC, USA) diluted 1:4000 in a blocking buffer. Plates were again incubated for 1 h at 37 °C and then washed 4 times with 200 µL PBS with 0.1% Tween 20. A total of 100 µL of 3,39,5,59-tetramethylbenzidene (TMB) reagent (ENN301; Thermo Scientific, Waltham, MA, USA) was added to each well and allowed to incubate at RT until visible staining of the positive control wells—usually 5 to 10 min. The reaction was stopped with the addition of 100 µL/well of TMB stop solution (5150-0021; SeraCare, Milford, MA, USA). The absorbance was then read at 450 nm using an EnVision plate reader.

The half-maximal effective concentration (EC_50_) values were defined as the compound concentration at which there was a 50% reduction in nucleoprotein expression (ELISA) or luciferase signal (replicons) relative to infected cells with DMSO alone (0% inhibition) and uninfected control cells (100% inhibition). RDV and ODV EC_50_ values were calculated from curve fits using nonlinear regression (GraphPad Prism v8.1.2). EC_50_ fold change for variants was calculated for each experiment, for comparison with the relevant WA1 reference. Fold change across all experiments was then averaged to obtain the final reported values. For each clinical isolate, experiments were performed twice with technical triplicates. The variability of the ELISA assay was estimated after repeat testing of the wildtype reference (RDV, n = 40; ODV, n = 11). The RDV EC_50_ values for the wildtype clinical isolate ranged between 44.2 nM and 196.8 nM (mean, 112.1 nM; median, 103.4 nM; standard deviation, 35.2 nM). Based on these data, we estimated that the assay variability was 2.8-fold. The ODV EC_50_ values for the wildtype clinical isolate ranged between 1.43 µM and 4.17 µM (mean, 2.29 µM; median, 2.11 µM; standard deviation, 0.75). Based on these data, we estimated that the assay variability was 2.9-fold. The variability of the subgenomic replicon assay was also estimated after repeated testing of the wildtype reference (RDV, n = 44; ODV, n = 36). The RDV EC_50_ values for the wildtype replicon ranged between 3.7 nM and 30.8 nM (mean, 11.3 nM; median, 10.1 nM; standard deviation, 4.6 nM); the estimated assay variability was 2.5-fold. The ODV EC_50_ values for the wildtype replicon ranged from 0.38 µM to 0.97 µM (mean, 0.62 µM; median, 0.61 µM; standard deviation, 0.14 µM); the estimated assay variability was 2.3-fold. Thus, any variant with fold change values below the respective threshold for RDV or ODV is within the variability of the assay.

For Omicron variants JN.1.7, JN.1.18, KP.2, KP.3, and LB.1, clinical isolates were not available; thus, a noninfectious SARS-CoV-2 replicon system (four DNA fragments isolated from SARS-CoV-2 SH01, SARS-CoV-2/human/CHN/SH01/2020, GenBank MT121215) was used to assess RDV and ODV antiviral activity. The established replicon method has been previously described [39] and used for phenotypic analysis of SARS-CoV-2 site-directed mutants of the replication complex components [40,41]. Briefly, the variant-defining substitutions identified in the replication complex genes (Nsp8 to Nsp14) were introduced into the luciferase-expressing replicon, the replicon was transfected into Huh7-1CN cells by electroporation, and cells were treated with 4-fold serial dilutions of RDV (8 dilutions total; 10 µM–0.6 nM) or ODV (8 dilutions total; 200 µM–12 nM). After 48 h, cell supernatants were collected, and luciferase signal was measured to determine EC_50_ values of each compound by curve fits using non-linear regression (GraphPad Prism v8.1.2). Two experiments were performed with technical triplicates.

To evaluate the prevalence of Nsp12 polymorphisms (≥1% of sequences) observed in Omicron variants of concern, VOIs, or VUMs, SARS-CoV-2 sequences from the Global Initiative on Sharing Avian Influenza Data EpiCoV database were evaluated. To assess the impact of Nsp12 polymorphisms on the antiviral activity of RDV and ODV, site-directed mutants bearing the identified Nsp12 polymorphisms were evaluated using the same replicon system described above.

Structural analysis of the identified polymorphisms was conducted on a composite model of cryo-electron microscopy structures of the replication-transcription complex retrieved from the Research Collaboratory for Structural Bioinformatics Protein Data Bank (ID#: 6XEZ and 7UO4) [42,43].

## 3. Results

### 3.1. Antiviral Activity of RDV and ODV Against Clinical Isolates of Omicron Variants

**Figure 1:**
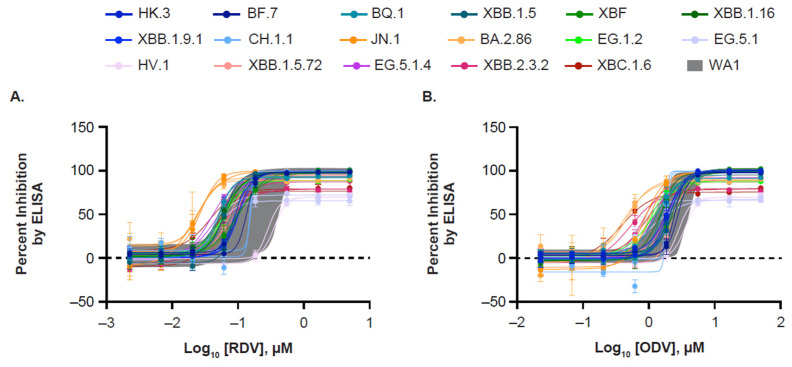
Dose–response curves of SARS-CoV-2 clinical isolates treated with RDV and ODV. A549-hACE2-TMPRSS2 cells were infected with the WA1 reference strain or Omicron variant clinical isolates at a multiplicity of infection of 0.05 and treated with 3-fold serial dilutions of (**A**) RDV (starting at 5 µM) or (**B**) ODV (starting at 50 µM). At 72 h post infection, an intracellular SARS-CoV-2 nucleoprotein antibody ELISA was used to detect viral replication and calculate percent inhibition by RDV and ODV. Each variant was tested two times with technical triplicates. The range of 24 replicates of the wildtype WA1 reference strain is shaded in gray. ELISA, enzyme-linked immunosorbent assay; ODV, obeldesivir; RDV, remdesivir.

Mean RDV EC_50_ values for all tested Omicron variants (BA.2.86, BF.7, BQ.1, CH.1.1, EG.1.2, EG.5.1, EG.5.1.4, FL.22, HK.3, HV.1, JN.1, XBB.1.5, XBB.1.5.72, XBB.1.16, XBB.2.3.2, XBC.1.6, and XBF) ranged from 21.8 nM (XBB.2.3.2) to 155 nM (CH.1.1), with a fold change range of 0.28 (JN.1) to 1.25 (BF.7) compared with the WA1 reference strain (Table 1, Figure 1). Mean ODV EC_50_ values ranged from 438 nM (BA.2.86) to 3193 nM (HV.1), with a fold change range of 0.23 (BA.2.86) to 1.24 (BF.7) compared with the WA1 reference strain (Table 1). All EC_50_ fold change values were within the variability of the assay. Overall, these results demonstrated that the tested Omicron variants remained as susceptible to RDV and ODV as the ancestral WA1 isolate.

### 3.2. Antiviral Activity of RDV and ODV Against Omicron Variants in the Replicon System

For Omicron variants JN.1.7, JN.1.18, KP.2, KP.3, and LB.1, clinical isolates were not available; thus, the variant-defining substitutions observed in the replication complex of these variants were introduced into a replicon by site-directed mutagenesis to assess RDV and ODV antiviral activity. When tested in replicons bearing the substitutions in the replication complex genes, mean EC_50_ values for RDV and ODV against these variants were 12.6 nM and 465 nM, respectively (Table 1). The EC_50_ fold changes were 1.14 for RDV and 1.05 for ODV compared with the wildtype reference replicon. All EC_50_ fold change values were within the variability of the assay, demonstrating no change in susceptibility.

### 3.3. Characterization of Nsp12 Polymorphisms Observed in Omicron Variants

Analysis of more than 2 million Omicron variant sequences revealed 29 polymorphisms in Nsp12 compared with the Wuhan reference (Wuhan-Hu-1 viral isolate; NC_045512). The Nsp12-defining amino acid substitutions (≥75% of sequences) included P323L, which was observed in all Omicron variants; Y273H, which was observed in BQ.1; G671S, which was observed in XBB.1.5, CH.1.1, and XBF; D63N, which was observed in HK.3; and G823insD, which was observed in XBC.1.6 (Table 1). Less prevalent polymorphisms were observed with frequencies ranging from 1.0% to 15.9% (T26I, D40G, T85I, G108D, D155G, I171V, Y175H, I223M, T225I, L247F, T248I, V257F, D258N, Y289H, D303N, T394M, P461S, V476A, N507I, A529V, M666I, F694Y, T803I, V848I; Table 1).

### 3.4. Impact of Nsp12 Polymorphisms on RDV and ODV Antiviral Activity in the Replicon System

Each substitution was introduced into the replicon system by site-directed mutagenesis, and mean RDV EC_50_ values ranged from 3.88 nM (T85I) to 18.0 nM (P461S), with fold changes ranging from 0.52 (L247F) to 1.60 (P461S, G671S) compared with the SH01 wildtype reference replicon (Table 1). Mean ODV EC_50_ values ranged from 212 nM (D155G) to 1322 nM (G671S), with fold changes ranging from 0.47 (D155G, V257F) to 1.76 (G671S) compared with the reference replicon. Altogether, this phenotypic analysis of Nsp12 polymorphisms showed no change in RDV or ODV susceptibility.

### 3.5. Structural Analysis of Identified Nsp12 Polymorphisms

**Figure 2:**
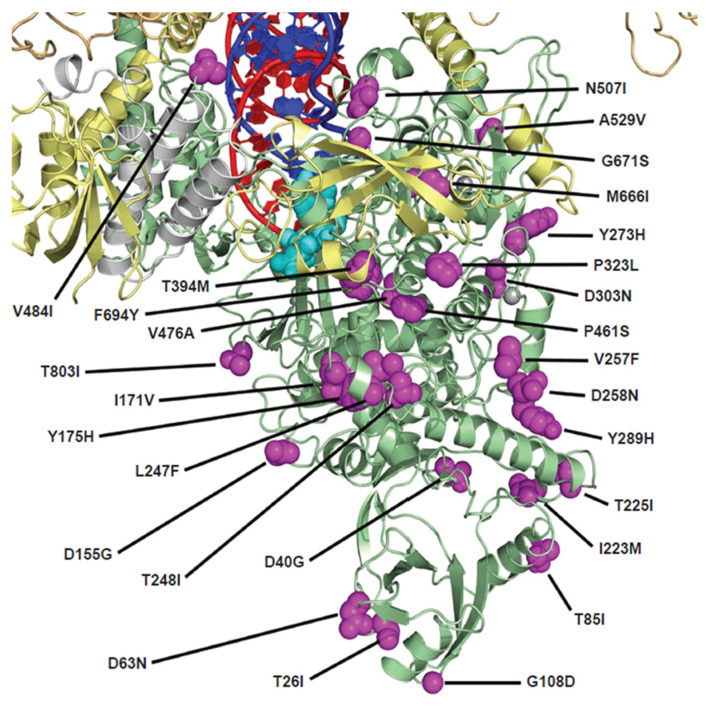
Map of Nsp12 amino acid polymorphisms observed in Omicron variants at ≥1% frequency. The structure is a model of the active NTP in the SARS-CoV-2 active site based on the cryo-electron microscopy structures 6XEZ [42] and 7UO4 [43]. The pre-incorporated active NTP metabolite of remdesivir and obeldesivir is shown in cyan. The Nsp12 protein is shown in green, with the locations of the observed polymorphisms shown in magenta. Other replication complex components, Nsp7, Nsp8 (two subunits), and Nsp13 (two subunits), are shown in white, yellow, and orange, respectively. The template RNA strand is shown in blue and the nascent RNA strand is shown in red. NTP, nucleoside triphosphate; RNA, ribonucleic acid.

None of the polymorphisms showed direct interaction with the incoming active NTP substrate or the viral RNA, except N507I, which interacts with the 5′ template overhang (Figure 2). Despite this interaction, phenotyping of the N507I replicon showed no reduction in susceptibility to RDV or ODV (Table 1).

## 4. Discussion

Since May 2024, the United States has observed a rise in COVID-19 activity, including increases in the rates of test positivity, hospitalizations, and deaths [5]. This resurgence is largely driven by the latest SARS-CoV-2 Omicron variants, including KP.2 and KP.3 [5], with growing concern that even more severe variants may be forthcoming. Among the associated public health challenges, the ongoing emergence of new variants poses a potential risk for changes in susceptibility to antiviral drugs. Therefore, it is crucial to continually test the susceptibility of new variants to widely used antiviral therapies, such as RDV, to ensure persistent treatment efficacy.

In this analysis, the in vitro antiviral activity of RDV and ODV, an oral prodrug of the parent nucleoside of RDV, against recent SARS-CoV-2 Omicron subvariants was assessed. RDV and ODV maintained activity against all tested Omicron variants, with potencies that were comparable with those observed when tested against the ancestral reference. Phenotyping of the 29 identified Nsp12 polymorphisms similarly showed no change in RDV or ODV antiviral activity relative to the SH01 reference strain.

These results are consistent with previous analyses wherein multiple Omicron variants that were distinct from those analyzed herein and earlier VOIs and VUMs remained susceptible to both compounds [9,38]. Together, these findings support continued use of RDV for the treatment of COVID-19 in the context of Omicron variants.

## Figures and Tables

**Table 1:** Phenotyping of Omicron variants and site-directed mutants.

	Substitutions in the Replication Complex ^a^		RDV		ODV	
			Mean EC_50_ ± SD (nM) ^b^	Fold Change ± SD ^c^ From Wildtype ^d^ Reference	Mean EC_50_ ± SD (nM) ^b^	Fold Change ± SD ^c^ From Wildtype ^d^ Reference
Variant lineage (clinical isolates) ^e^						
SARS-CoV-2 reference strain (WA1; lineage A) ^f^	–		Range, 58–165	1.00	Range, 1240–4125	1.00
BA.2.86 ^f^	Nsp9 T35I Nsp12 P323L Nsp13 R392C Nsp14 I42V		26.0 ± 0.3	0.36 ± 0.04	438 ± 52	0.23 ± 0.03
BF.7 ^f^	Nsp12 P323L Nsp13 R392C Nsp14 I42V		120 ± 24	1.25 ± 0.35	2644 ± 230	1.24 ± 0.04
BQ.1 ^f^	Nsp12 P323L Y273H Nsp13 M233I R392C Nsp14 I42V		50.8 ± 2.4	0.53 ± 0.02	1410 ± 113	0.66 ± 0.02
CH.1.1 ^f^	Nsp8 N118S Nsp12 P323L G671S Nsp13 R392C Nsp14 I42V V182I		155 ± 4	0.95 ± 0.03	1835 ± 50	0.62 ± 0.16
EG.1.2 ^g^	Nsp9 T35I Nsp12 P323L G671S Nsp13 S36P R392C Nsp14 I42V		59.9 ± 0.2	0.87 ± 0.23	1090 ± 57	0.86 ± 0.03
EG.5.1 ^g^	Nsp9 T35I Nsp12 P323L G671S Nsp13 S36P R392C Nsp14 I42V		44.4 ± 0.6	0.58 ± 0.03	903 ± 243	0.47 ± 0.04
EG.5.1.4 ^h^	Nsp9 T35I Nsp12 P323L G671S Nsp13 S36P R392C Nsp14 I42V		44.3 ± 7.5	0.40 ± 0.06	1473 ± 379	0.41 ± 0.01
FL.22 ^i^	Nsp9 T35I Nsp12 P323L Nsp13 S36P R392C Nsp14 I42V		96.9 ± 10.0	1.15 ± 0.01	2070 ± 28	0.88 ± 0.03
HK.3 ^g^	Nsp9 T35I Nsp12 D63N P323L G671S Nsp13 S36P R392C Nsp14 I42V		93.3 ± 1.3	1.04 ± 0.15	1921 ± 1	1.03 ± 0.24
HV.1 ^h^	Nsp9 T35I Nsp12 P323L G671S Nsp13 R392C Nsp14 I42V V182I		135 ± 6	0.93 ± 0.02	3193 ± 513	0.92 ± 0.34
JN.1 ^f^	Nsp9 T35I Nsp12 P323L Nsp13 R392C Nsp14 I42V		25.0 ± 3.8	0.28 ± 0.04	912 ± 99	0.31 ± 0.02
XBB.1.5 ^f^	Nsp12 P323L G671S Nsp13 S36P R392C Nsp14 I42V		76.7 ± 18.7	0.81 ± 0.26	2249 ± 143	1.06 ± 0.13
XBB.1.5.72 ^h^	Nsp12 P323L G671S Nsp13 S36P R392C Nsp14 I42V		82.8 ± 10.8	0.75 ± 0.09	2271 ± 66	0.66 ± 0.17
XBB.1.16 ^i^	Nsp12 P323L G671S Nsp13 S36P R392C Nsp14 I42V D222Y		60.4 ± 5.7	0.73 ± 0.15	1475 ± 35	0.63 ± 0.01
XBB.2.3.2 ^i^	Nsp12 P323L G671S Nsp13 R392C Nsp14 I42V		21.8 ± 4.8	0.29 ± 0.10	671 ± 205	0.30 ± 0.03
XBC.1.6 ^i^	Nsp12 P323L G671S G823insD Nsp13 P77L T127I I33V		40.9 ± 4.7	0.46 ± 0.06	638 ± 352	0.26 ± 0.13
XBF ^i^	Nsp8 N118S Nsp12 P323L G671S Nsp13 R392C Nsp14 I42V V182I		102 ± 4	1.22 ± 0.09	2345 ± 7	0.99 ± 0.05
Variant lineage (replicon system) ^j^						
SARS-CoV-2 reference (SH01; lineage B)	–		Range, 10.9–11.0	1.00	Range, 375–526	1.00
JN.1.7 JN.1.18 KP.2 KP.3 LB.1	Nsp9 T35I Nsp12 P323L Nsp13 S36P R392C Nsp14 I42V		12.6 ± 1.0	1.14 ± 0.09	465 ± 45	1.05 ± 0.15
	**Omicron lineage**	**Polymorphism** **frequency,** **% (n)**				
Nsp12 polymorphism (replicon system)						
SARS-CoV-2 reference (SH01; lineage B)	B	–	Range, 4.4–13.1	1.00	Range, 390–965	1.00
T26I	BA.2.86	1.3 (10) ^k^	6.39 ± 0.12	1.23 ± 0.06	856 ± 41	1.17 ± 0.002
D40G	BQ.1	1.2 (624) ^k^	13.3 ± 3.2	1.16 ± 0.20	690 ± 90	1.20 ± 0.31
D63N	EG.5.1	2.6 (699) ^k^	15.1 ± 0.1	1.25 ± 0.14	791± 42	1.23 ± 0.06
	HK.3	99.8 (30,557) ^k^				
T85I	EG.5.1	1.1(305) ^k^	3.88 ± 0.21	0.87 ± 0.06	839 ± 3	1.17 ± 0.05
G108D	BJ.1	1.8 (3) ^k^	6.85 ± 0.36	0.81 ± 0.03	Not tested	NA
D155G	BA.2.3.20	2.9 (25) ^l^	7.94 ± 0.33	1.39 ± 0.11	212 ± 32	0.47 ± 0.10
I171V	EG.5	1.0 (14) ^m^	6.01 ± 2.04	1.19 ± 0.44	633 ± 87	1.11 ± 0.10
Y175H	CH.1.1	1.2 (237) ^k^	5.60 ± 0.12	1.10 ± 0.06	690 ± 31	1.21 ± 0.004
I223M	XBB.1.9.1	1.9 (329) ^k^	17.2 ± 1.3	1.52 ± 0.22	767 ± 35	1.33 ± 0.23
T225I	DV.7	10.5 (39) ^m^	8.00 ± 0.12	1.53 ± 0.12	1022 ± 51	1.39 ± 0.002
L247F	BF.7	3.1 (1661) ^k^	5.95 ± 0.94	0.52 ± 0.15	681 ± 50	0.80 ± 0.19
T248I	BF.7	2.0 (1292) ^n^	5.33 ± 0.58	0.93 ± 0.21	387 ± 47	0.85 ± 0.16
V257F	XBB.1.5	3.1 (4819) ^n^	6.84 ± 0.07	1.19 ± 0.16	215 ± 9	0.47 ± 0.05
D258N	XBB.1.9.2	1.0 (93) ^k^	12.0 ± 5.6	1.01 ± 0.56	817 ± 11	1.27 ± 0.01
Y273H	BQ.1	98.0 (52,625) ^k^	5.74 ± 0.03	1.00 ± 0.13	228 ± 4	0.50 ± 0.03
Y289H	XBB.2.3	1.4 (125) ^k^	15.8 ± 4.1	1.32 ± 0.47	987 ± 121	1.54 ± 0.20
D303N	BA.2.86	15.9 (130) ^o^	15.9 ± 0.5	1.40 ± 0.14	563 ± 10	0.97 ± 0.10
P323L	Across Omicron subvariants	98.8 (16,265,492) ^k^	7.21 ± 0.18	0.94 ± 0.03	333 ± 32	0.78 ± 0.19
T394M	BA.2.86	1.8 (15) ^o^	5.42 ± 0.12	1.03 ± 0.08	574 ± 18	1.06 ± 0.12
P461S	XBB.1.9.1	1.7 (209) ^k^	18.0 ± 5.5	1.60 ± 0.60	641 ± 206	1.08 ± 0.21
V476A	EG.5	1.0 (8) ^p^	6.68 ± 0.66	0.55 ± 0.002	329 ± 49	0.51 ± 0.08
N507I	XBF	1.8 (176) ^k^	6.29 ± 0.52	0.85 ± 0.11	369 ± 16	0.67 ± 0.01
A529V	BF.7	1.3 (693) ^k^	Did not replicate	NA	Did not replicate	NA
M666I	XBB.1.9.1	1.4 (249) ^q^	9.83 ± 2.12	0.80 ± 0.09	603 ± 193	0.94 ± 0.30
G671S	XBB.1.5	95.7 (177,617) ^k^	15.5 ± 1.1	1.60 ± 0.10	1322 ± 17	1.76 ± 0.09
	CH.1.1	92.5 (18,857) ^k^				
	XBF	97.0 (9552) ^k^				
F694Y	XBF	2.4 (239) ^k^	Did not replicate	NA	Did not replicate	NA
T803I	XBB.2.3	1.5 (75) ^r^	6.31 ± 0.002	0.56 ± 0.04	370 ± 37	0.64 ± 0.15
G823insD	XBC.1.6	89.7 (1155) ^k^	6.30 ± 1.50	0.75 ± 0.17	740 ± 17	1.03 ± 0.07
V848I	XBB.1.16	2.5 (900)^m^	13.4 ± 2.3	1.11± 0.30	847 ± 70	1.32 ± 0.10

BEI, Biodefense and Emerging Infectious Research; EC_50_, half-maximal effective concentration; GISAID, Global Initiative on Sharing Avian Influenza Data; NA, not applicable; ODV, obeldesivir; RDV, remdesivir; SD, standard deviation. ^a^ The characteristic substitutions for a lineage are defined as nonsynonymous substitutions or deletions that occur in ≥75% of sequences within that lineage. ^b^ EC_50_ ± SD values represent the mean of ≥2 independent experiments. ^c^ Fold change ± SD represents the mean of ≥2 independent experiments. Fold change was calculated for each experiment, from which the mean fold change was calculated. ^d^ For clinical isolates, the wildtype SARS-CoV-2 reference strain was the WA1 strain (lineage A). For the replicon system, the wildtype SARS-CoV-2 reference strain was the SH01 replicon (lineage B). ^e^ The clinical isolates used in this study were: BF.7 (hCoV-19/USA/MD-HP38288/2022; NR-58974; deposited to BEI by Dr. Andrew Pekosz), BQ.1 (hCoV-19/USA/MD-HP38960/2022; NR-58975; deposited to BEI by Dr. Andrew Pekosz), XBB.1.5 (hCoV-19/USA/MD-HP40900/2022; NR-59104; deposited to BEI by Dr. Andrew Pekosz), CH.1.1 (hCoV-19/USA/MD-HP41275/2022; NR-59204; deposited to BEI by Dr. Andrew Pekosz), XBF (hCoV-19/Japan/TY41-831/2022; TY41-831), XBB.1.6 (hCoV-19/Japan/TY41-984/2023; TY41-984), FL.22 (hCoV-19/Japan/TY41-951/2023; TY41-951), JN.1 (hCoV-19/USA/New York/PV96109/2023; NR-59693), XBB.2.3.2 (hCoV-19/Japan/TY42-006/2023), EG.5.1 (hCoV-19/USA/MD-HP47946), EG.1.2 (hCoV-19/USA/MD-HP46933-PIDKWGVVMR/2023), XBC.1.6 (hCoV-19/Japan/TY42-004/2023), HK.3 (hCoV-19/USA/MD-HP49292-PIDWKNSGGH/2023), BA.2.86 (hCoV-19/USA/MI-UM-10052670540/2023; NR-59638; deposited to BEI by the Centers for Disease Control and Prevention), HV.1 (hCoV-19/USA/CA-GS137943/2024), XBB.1.5.72 (hCoV-19/USA/CA-GS136867/2024), and EG.5.1.4 (hCoV-19/USA/CA-GS138000/2024). ^f^ Sourced from BEI Repository. ^g^ Sourced from Johns Hopkins University. ^h^ Sourced from Gilead Sciences, Inc. ^i^ Sourced from National Institute of Infectious Diseases, Japan. ^j^ EC_50_ values from variants tested using the replicon system cannot be directly compared with variants tested using clinical isolates due to differences in assay reagents, conditions, and detection technology. Fold change values, which are independent of the assay used, offer a more accurate basis for comparison. ^k^ Frequency of the observed polymorphism in the GISAID database as of 7 August 2024. ^l^ Was observed at ≥1% frequency (2.9%, D155G) in the GISAID database on 28 November 2022, but has since decreased below 1%. ^m^ Was observed at ≥1% frequency (1.0%, I171V; 10.5%, T225I; 2.5%, V848I) in the GISAID database on 10 August 2023 but has since decreased below 1%. ^n^ Was observed at ≥1% frequency (2.0%, T248I; 3.1% V257F) in the GISAID database on 6 April 2023 but has since decreased below 1%. ^o^ Was observed at ≥1% frequency (15.9%, D303N; 1.8%, T394M) in the GISAID database on 4 March 2024 but has since decreased below 1%. ^p^ Was observed at ≥1% frequency (1.0%, V476A) in the GISAID database on 3 October 2023 but has since decreased below 1%. ^q^ Was observed at ≥1% frequency (1.4%, M666I) in the GISAID database on 1 June 2023 but has since decreased below 1%. ^r^ Was observed at ≥1% frequency (1.5%, T803I) in the GIAID database on 5 September 2023 but has since decreased below 1%.

## Data Availability

The raw data supporting the conclusions of this article will be made available by the authors upon request.
